# Modeling the behavior of a generalized Cholera epidemic model with asymptomatic measures for early detection

**DOI:** 10.1371/journal.pone.0319684

**Published:** 2025-03-31

**Authors:** Ali Hasan Ali, Aqeel Ahmad, Fakher Abbas, Evren Hincal, Abdul Ghaffar, Belal Batiha, Husam A Neamah

**Affiliations:** 1 Department of Mathematics, College of Education for Pure Sciences, University of Basrah, Basrah, Iraq; 2 Jadara University Research Center, Jadara University, Irbid, Jordan; 3 Department of Mathematics, Ghazi University, Dera Ghazi Khan, Pakistan; 4 Mathematics Research Center, Near East University, Near East Boulevard, Nicosia North Cyprus, Turkey; 5 Department of Mathematics, Near East University, Near East Boulevard, Nicosia North Cyprus, Turkey; 6 Mathematics Department, Faculty of Science and Information Technology, Jadara University, Irbid, Jordan; 7 Department of Electrical Engineering and Mechatronics, Faculty of Engineering, University of Debrecen, Debrecen, Hungary; 8 Technical Engineering College, Al-Ayen University, Dhi Qar, Iraq; 9 Department of Business Management, Al-imam University College, Balad, Iraq; Princess Sumaya University for Technology, JORDAN

## Abstract

To understand how a disease spreads through a society, mathematical formulations are a crucial tool for comprehending the complete dynamics of cholera. Model formulations are essential for thoroughly understanding the propagation of cholera throughout a population. For an assessment of the stable state of a newly established SEIRB system, both qualitative and quantitative evaluations are conducted. The reproductive number is derived to observe the infection spread rate among patients. Additionally, sensitivity analyses are performed to assess the impact of each parameter and to determine the rate of change in each. The existence of positive solutions with linear growth has been verified using global derivatives, and the level of effect in each subsection is determined through the application of Lipschitz criteria. By employing Lyapunov’s first derivative of the function, the framework is analyzed for global stability to evaluate the overall effect of both symptomatic and asymptomatic measures following early detection interventions. The Mittag-Leff1er kernel is utilized to obtain a robust solution via a fractal-fractional operator, enabling continuous monitoring for improved control measures. Simulations are performed to assess the global impact of both symptomatic and asymptomatic consequences of cholera and to observe the actual behavior of the disease. It has been confirmed that individuals with strong immune systems will recover efficiently if the infection is diagnosed early through timely detection measures. This analysis provides insight into the current state of cholera control, comparing outcomes for those receiving treatment and those whose robust immune systems negate the need for medication. Such investigations will enhance our understanding of disease transmission and support the development of effective control strategies based on our validated findings.

## 1 Introduction

Since Fibonacci introduced the well-known Fibonacci series to model population growth in the early 12th century, mathematics has been an integral part of biology [1]. Daniel Bernoulli used mathematical concepts to demonstrate their application to microscopic objects. In 1901, Johannes Ranke [2] coined the term ’biomathematics.’ The primary objective of biomathematics is to theoretically examine mathematical models to uncover the laws governing the growth and behavior of biological systems [3]. It aims to help us understand the complexities of living organisms. Mathematics has made significant contributions to the natural sciences, and it can be equally useful in advancing our understanding of the biological sciences [4]. For this reason, it is crucial to begin educating students about the interrelated aspects of mathematical biology early, starting with foundational knowledge [5]. An investigation in mathematical biology can be structured into several steps [6]. The first step involves presenting biological techniques that may raise additional biological questions, which mathematics could help address. The second step aims to explain a mathematical procedure that can be used to characterize a suitable biological model. The next step involves implementing mathematical models and additional techniques to apply them in the formulation of mathematical laws. The final step is to draw conclusions regarding the mathematical results within the context of the biological methods used.

Aspects of fractional calculus (FC) [7] can be useful for solving a wide range of scientific problems. Various operators, including Caputo [8], Grünwald-Letnikov, Riemann-Liouville, Fabrizio (CF), Caputo-Fabrizio [9], and Atangana-Baleanu [10], have been explained. The bacterium Vibrio cholerae is responsible for the severe intestinal disease cholera [11]. The fecal-oral route is the primary mode of transmission, where infectious bacteria are passed from an infected person to another, typically through vomit or feces [12]. This disease causes severe diarrhea and vomiting when contaminated food or water is ingested [13]. For many years, cholera has raised serious concerns regarding hygiene and the lack of social infrastructure, even in aff1uent countries.

Cholera remains a global public health concern due to recent outbreaks in Zimbabwe [14], Tanzania [15], Ethiopia [16], Kenya [17], Yemen [18], and other nations. A combination of human factors, climate, and microbial agents contributes to the spread of cholera [19]. It spreads both directly through human contact and indirectly through climate-related pathways. Cholera has had a significant impact and has been widely studied for its effects on public health and economic development. In theory and practice, cholera can be controlled by taking appropriate measures, such as treating infected individuals and maintaining good hygiene. Over time, efforts can be made to develop effective prevention and treatment strategies

Toxic diseases such as cancer have spread globally in recent years, affecting all levels of society (see [20]). The complex mechanisms behind the spread of cholera have been explored and understood [21]. In [22], an optimal control mechanism for cholera epidemics was developed in a mathematical model and analyzed using Pontryagin’s maximum principle. Experts emphasized immunizations, medical care, and public education programs as key strategies for controlling widespread cholera outbreaks. However, the model did not include a safe household water source as a control parameter. A model was developed in Zimbabwe between 2008 and 2009 to examine the spread of cholera, according to the authors [23]. Cholera epidemics in Africa highlight the importance of the person-to-person (p-to-p) transmission channel. The model considers both direct (p-to-p) and indirect (environment-to-person, e-to-p) transmission routes. The analysis in [24] modified the proposed cholera model, while [25] investigated the most effective intervention strategies and other control options; however, neither took human infection into account. In Tanzania, researchers modified and studied a deterministic cholera model by incorporating human education campaigns along with water treatment and control technologies. In the analysis of this model, they did not conduct a quantitative assessment of the basic reproduction number, which is essential for understanding disease transmission. The authors of [26] presented a mathematical model of cholera, in which public health measures serve as the primary cholera control strategies.

Additionally, some authors utilize different fractional operators to investigate various physical phenomena. For example, authors employ the Caputo operator, which significantly advanced fractional-order differential theory after the Riemann-Liouville operator [27,28]. Different fractional-order operators have been applied to physical problems in biological and engineering systems [29]. These fractional-order operators reflect the genetic and behavioral aspects of memory present in biological and engineering systems [30]. It is well-known that integer-order operators cannot capture memory effects as effectively as fractional-order operators, which can model these effects in geographical systems, even in the absence of external variables [31]. Moreover, for various disease models, fractional derivatives provide more accurate predictions compared to actual data; see [32]. The computation of solutions for corruption systems is performed using the Power-Law, Mittag-Leff1er, and Exponential Decay kernels via fractional derivatives [33]. A mathematical model for boosting the immune system was developed in [34] and transformed into a fractional-order model through the application of the Caputo fractional operator. Another immune system-boosting mathematical model was created and then transformed into a fractional-order model using the ABC operator [35]. The goal of that work was to investigate the use of cytokines and anti-PD-L1 inhibitors in the diagnosis and treatment of lung cancer in individuals with compromised immune systems [36]. The fractional-order derivatives in studies on cholera models account for the impact of personal hygiene practices, travel, and treatment of affected individuals. Several other related studies have investigated liver cirrhosis caused by HBV with early detection and chemotherapy [37], the stabilizing effect of small prey immigration in predator-prey systems [38], and the influence of psychological panic, glucose risk, and estrogen on breast cancer dynamics [39].

We conducted research on cholera using an innovative approach to effectively control the disease, particularly in populations that are both protected and infected. The main goal of this work is to develop a new mathematical model for the recovery effect that incorporates early detection and control methods for cholera. Cholera poses a serious threat to human life. To aid readers in understanding the innovation, Section 1 provides an introduction and historical background, including the basic definitions used in the subsequent research. In Section 2, a new mathematical model is introduced for the recovery effect under the proposed hypothesis, along with control measures. Section 3 discusses the analysis of the cholera model, focusing on positivity, boundedness, and the study of positive solutions using non-local kernels. Additionally, qualitative and quantitative analyses, reproduction number analysis, and sensitivity analysis are presented. Section 4 examines the impact of the global derivative using the Riemann-Stieltjes integral and norm. The global stability of the model is addressed in Section 5, employing Lyapunov’s derivative for data analysis. Numerical solutions are constructed in Section 6, utilizing an ML kernel and a fractional operator. Section 7 outlines the simulation, which provides a comprehensive physical understanding and was implemented using MATLAB code. Finally, the conclusion is given in Section 8.

### 1.1 Basic definitions

**Definition 1** For 0 ≤ *ℓ* , *ı* ≤ 1, the function *∏* ⁡ ( *t* )  in the Riemann-Liouville fractional operator with a generalized Mittag-Leff1er kernel is specified as follows:


$$\begin{array}{cccc} {}^{FFM}D_{0,t}^{\ell ,ı}(\prod (t))=\frac{AB(\ell )}{1-\ell }\frac{d}{dt^{ı}}\overset{t}{\underset{0}{\int }}E_{\ell }\left[-\frac{\ell }{1-\ell }{\left(t-\varsigma \right)}^{\ell }\prod (\varsigma )\right]d\varsigma , & & & \end{array}$$

where 0 < *ℓ* , *ı* ≤ 1 and $AB(\ell )=1-\ell +\frac{\ell }{\Gamma (\ell )}.$ Thus, *∏* ⁡ ( *t* )  with order  ( *ℓ* , *ı* )  and having Mittag-Leff1er type kernel is defined as:


$$\begin{array}{cccc} {}^{FFM}D_{0,t}^{\ell ,ı}(\prod (t)) & = & \frac{ı(1-\ell )t^{ı-1}\prod (t)}{AB(\ell )}+\frac{\ell ı}{AB(\ell )}\int_{0}^{t}\varsigma^{\ell -1}(t-\varsigma )\prod (\varsigma )d\varsigma . & \end{array}$$

## 2 Formulation of SEIRB generalized Cholera model

In this paper, we employed differential equations for the constructed model under created hypothesis of early detection and vibrio bacteria spread in the environment, which also includes a Fractal-Fractional order derivative operator for continuous monitoring of spread for better control of the cholera epidemic. The portions of the recommended investigation are used to highlight the relationship between an individual’s physical health state and the level of Vibrio bacteria present in their surroundings. The straight lines depicts movement while dotted lines depicts contribution of the bacteria and can be seen in flow diagram. The ensuing presumptions serve as the foundation for the proposed and investigated mathematical model:

1. We divided the analysis into 4 subcategories for human beings: *S*, *E*, *I*, and *R* indicate the people who are susceptible, people who were exposed treated as early detection in which symptom does not appear but infection exist at acute stage and are recovered without medication, people who were infectious need proper medication and are considered symptomatic individuals, and people who were recovered for both asymptomatic and symptomatic individuals, respectively. Therefore, *N*(*t*) indicates the entire population of humans. It is described as follows, *N* = *S* + *E* + *I* + *R*; *B* is the amount of Vibrio bacteria present in the surrounding environment which causes environmental effects. The purpose is to improve the immune system at asymptomatic stage by early detection measures to stop to become infectious as symptomatic stage. Also to reduce the vibrio bacterial infection from the environment release by cholera infected individuals.
2. Parameters and variables are defined as follows and are presumed to be non-negative throughout the document: The new recruitment rate and the natural mortality rate of humans are represented by the symbols  ∝  and *ω*, respectively; these indicate the average amount of time that individuals spend during the infectious phase.
3. People are linked to the percentage of recovered-class people who didn’t lose their understanding or immunity throughout the epidemic of cholera, and it is expected that people learn about the disease through instruction and advertising. On another note, patients who do not take treatment for themselves ultimately pass away at a rate known as $\omega_{1}$ from their disease, whereas patients who receive a standard rate of disease recovery known as *ϕ* are achieved with efficient medical treatment.
4. The parameters *μ* and *ν* represent the level of infection and expansion of Vibrio bacteria, the pace of patient recovery, the dispersal of Vibrio bacteria in their environment, and the propagation of exposed people. The parameters $\beta_{h}$ and $\beta_{e}$ represent the effects of infection associated with the propagation of transmission from person to person and from the environment to human beings, respectively.

**Fig 1:**
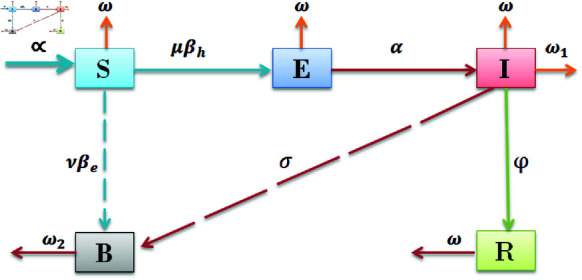
Flow Chart. The model formulation is shown in the flow chart.

The flow chart according to our created hypothesis is shown in Figure 1.

So, the mathematical form of the generalized cholera disease model by taking asymptomatic measures, as follows.


$$\begin{array}{c} \begin{array}{ccc} \frac{d}{dt}S(t)= & \propto -\mu \beta_{h}S(t)E(t)-\beta_{e}\nu S(t)B(t)-\omega S(t), & \\ \frac{d}{dt}E(t)= & \mu \beta_{h}S(t)E(t)-\left(\alpha +\omega \right)E(t),\\ \frac{d}{dt}I(t)= & \alpha E(t)-(\phi +\omega +\omega_{1})I(t),\\ \frac{d}{dt}R(t)= & \phi I(t)-\omega R(t),\\ \frac{d}{dt}B(t)= & \nu \beta_{e}B(t)S(t)+\sigma I(t)-\omega_{2}B(t). \end{array} \end{array}$$ (1)

The system indicated above is consistent with the initial conditions. $S^{0}=S(0),E^{0}=E(0),I^{0}=I(0),B^{0}=B(0),R^{0}=R(0)$.

Now, applying the definition of the Fractal-Fractional operator with the Mittag-Leff1er kernel, we obtain the model presented below:


$$\begin{array}{c} \begin{array}{ccc} {}_{0}^{FFM}D_{t}^{\xi ,\tau }S(t)= & \;\propto -\;\beta_{h}\;\mu E(t)S(\;t)-\;\nu \beta_{e}S(\;t)B(\;t)-\omega S(\;t), & \\ {}_{0}^{FFM}D_{t}^{\xi ,\tau }E(\;t)= & \;\mu \beta_{h}S(t)E(t)-(\alpha +\omega )E(t),\\ {}_{0}^{FFM}D_{t}^{\xi ,\tau }I(\;t)= & \;\alpha E(\;t)-(\phi +\omega +\omega_{1})I(\;t),\\ {}_{0}^{FFM}D_{t}^{\xi ,\tau }R(\;t)= & \;\phi I(\;t)-\omega R(\;t),\\ {}_{0}^{FFM}D_{t}^{\xi ,\tau }B(\;t)= & \;\sigma I(\;t)+\nu \beta_{e}S(\;t)B(\;t)-\omega_{2}B(\;t). \end{array} \end{array}$$ (2)

Considering initial conditions matching the system defined above $\;S^{0}=\;S(0)$, $\;E^{0}=\;E(0)$, $\;I^{0}=\;I(0)$, $B^{0}=B(0)$, $\;R^{0}=\;R(0)$.

## 3 Analysis of SEIRB generalized Cholera model

### 3.1 Positiveness and boundedness of generalized Cholera model

We Search regarding the criteria that secure positive outcomes of the suggested model’s solutions to show that they are appropriate and limited, presuming that they consist of actual circumstances with pertinent values. For this, we have


$$\begin{array}{c} S(t)\geq S^{0}e^{-(\mu \beta_{h}|E{|}_{\infty }+\nu \beta_{e}|B{|}_{\infty }+\omega )t},\forall t\geq 0 \end{array}$$ (3)

and the remaining equations will be


$$\begin{array}{c} E(t)\geq E^{\;0}e^{-\left(\omega +\alpha \right)t},\forall t\geq 0 \end{array}$$ (4)

$$\begin{array}{c} I(t)\geq I^{\;0}e^{-(\phi +\omega +\omega_{1})t},\forall t\geq 0 \end{array}$$ (5)

$$\begin{array}{c} R(t)\geq R^{\;0}e^{-(\omega )t},\forall t\geq 0 \end{array}$$ (6)

$$\begin{array}{c} B(t)\geq B^{\;0}e^{(-\omega_{2})t},\forall t\geq 0 \end{array}$$ (7)

Define the norm


||κ||∞= sup ⁡ t∈Dκ|κ(t)| (8)

so the $D_{\kappa }$ is the domain of *κ*. employing the Norm, we get for function *S*(*t*);


$$\begin{array}{rcll} {}_{0}^{FFM}D_{t}^{\xi ,\tau }S & = & \;\propto -\;\beta_{h}\;\mu ES-\nu \beta_{e}SB-\omega S, & \\ & \geq & -\beta_{h}\mu ES-\nu \beta_{e}SB-\omega S, & \\ & \geq & -(\beta_{h}\mu E+\nu \beta_{e}B+\omega )S, & \\ & = & -(\beta_{h}\mu ∥E{∥}_{\infty }+\nu \beta_{e}∥B{∥}_{\infty }+\omega )S, & \end{array}$$

For ordinary derivative, we have


$$\begin{array}{rcll} S & = & S_{0}e^{-(\beta_{h}\mu ∥E{∥}_{\infty }+\nu \beta_{e}∥B{∥}_{\infty }+\omega )t},\;\;\;\;\;\;\forall t\geq 0. & \end{array}$$

Positive outcomes utilizing a non-local operator are detailed in the following.

### 3.2 Positive solutions with non-local operator

For non-local operators, all outcomes of system (1) are positive [40] if all initial conditions are met.

- With a power law kernel for the Fractal-Fractional operator, we have *∀* ⁡ *t* ≥ 0.
  $$\begin{array}{rcll} S & \geq & S^{0}E_{\xi }\left(-\varphi^{1-\tau }(\mu \beta_{h}|E{|}_{\infty }+\nu \beta_{e}|B{|}_{\infty }+\omega )t^{\xi }\right), & \\ E & \geq & E^{0}E_{\xi }\left(-\varphi^{1-\tau }(\omega +\alpha )t^{\xi }\right), & \\ I & \geq & I^{0}E_{\xi }\left(-\varphi^{1-\tau }(\phi +\omega +\omega_{1})t^{\xi }\right), & \\ R & \geq & R^{0}E_{\xi }\left(-\varphi^{1-\tau }(\omega )t^{\xi }\right), & \\ B & \geq & B^{0}E_{\xi }\left(-\varphi^{1-\tau }(\omega_{2})t^{\xi }\right). & \end{array}$$
  Where the time component is *φ*.
- We obtain *∀* ⁡ *t* ≥ 0 for an operator with an exponential kernel that is fractal-fractional.
  $$\begin{array}{rcll} S & \geq & S^{0}exp\left(-\frac{\chi^{1-\tau }\xi (\mu \beta_{h}|E{|}_{\infty }+\nu \beta_{e}|B{|}_{\infty }+\omega )t}{M(\xi )-(1-\xi )\left[\mu \beta_{h}|E{|}_{\infty }+\nu \beta_{e}|B{|}_{\infty }+\omega \right]}\right), & \\ E & \geq & E^{0}exp\left(-\frac{\chi^{1-\tau }\xi (\omega +\alpha )t}{M(\xi )-(1-\xi )\left[\omega +\alpha \right]}\right), & \\ I & \geq & I^{0}exp\left(-\frac{\chi^{1-\tau }\xi (\phi +\omega +\omega_{1})t}{M(\xi )-(1-\xi )\left[\phi +\omega +\omega_{1}\right]}\right), & \\ R & \geq & R^{0}exp\left(-\frac{\chi^{1-\tau }\xi (\omega )t}{M(\xi )-(1-\xi )[\omega ]}\right), & \\ B & \geq & B^{0}exp\left(-\frac{\chi^{1-\tau }\xi (\omega_{2})t}{M(\xi )-(1-\xi )\left[\omega_{2}\right]}\right). & \end{array}$$
- With a Mittag-Leff1er kernel for the Fractal-Fractional operator, we have *∀* ⁡ *t* ≥ 0.
  $$\begin{array}{rcll} S & \geq & S^{0}E_{\xi }\left(-\frac{\chi^{1-\tau }\xi (\mu \beta_{h}|E{|}_{\infty }+\nu \beta_{e}|B{|}_{\infty }+\omega )t}{AB(\xi )-(1-\xi )\left[\mu \beta_{h}|E{|}_{\infty }+\nu \beta_{e}|B{|}_{\infty }+\omega \right]}\right), & \\ E & \geq & E^{0}E_{\xi }\left(-\frac{\chi^{1-\tau }\xi (\omega +\alpha )t}{AB(\xi )-(1-\xi )\left[\omega +\alpha \right]}\right), & \\ I & \geq & I^{0}E_{\xi }\left(-\frac{\chi^{1-\tau }\xi (\phi +\omega +\omega_{1})t}{AB(\xi )-(1-\xi )\left[\phi +\omega +\omega_{1}\right]}\right), & \\ R & \geq & R(0)E_{\xi }\left(-\frac{\chi^{1-\tau }\xi (\omega )t}{AB(\xi )-(1-\xi )[\omega ]}\right), & \\ B & \geq & B^{0}E_{\xi }\left(-\frac{\chi^{1-\tau }\xi (\omega_{2})t}{AB(\xi )-(1-\xi )\left[\omega_{2}\right]}\right). & \end{array}$$

### 3.3 Qualitative and quantitative analysis

The feasible equilibrium of cholera model will be given in this part.

**Theorem 1:** To ensure the existence of an equilibrium in the generalized cholera model, the following statement must hold:

1. The cholera model’s disease free point, $F_{S0000}(\frac{\propto }{\omega },0,0,0,0)$ *∀* ⁡  $\mu ,\propto ,\nu ,\omega ,\omega_{1},\omega_{2},\beta_{h},\beta_{e},\sigma ,$ *α* ,  *ϕ* > 0 .
2. The cholera model endemic point, $F_{SEIRB}^{+}(S^{*},E^{*},I^{*},R^{*},B^{*})$.

**Proof:** Epidemic and endemic equilibrium points are two distinct types of equilibrium points. To find them, the right-hand sides of the equations associated with the system are set to be “0". If there is no cholera spread in the population of $F_{0}$, the constant production is the disease-free equilibrium point. Now, by setting


$${}_{0}^{FFM}D_{t}^{\xi ,\tau }S{=}_{0}^{FFM}D_{t}^{\xi ,\tau }E{=}_{0}^{FFM}D_{t}^{\xi ,\tau }I{=}_{0}^{FFM}D_{t}^{\xi ,\tau }R{=}_{0}^{FFM}D_{t}^{\xi ,\tau }B=0,$$

and right-hand sides of system to be zero, we get


$$\begin{array}{rcll} 0 & = & \propto -\mu \beta_{h}ES-\nu \beta_{e}BS-\omega S, & \\ 0 & = & \mu \beta_{h}ES-\left(\alpha +\omega \right)E, & \\ 0 & = & \alpha E-(\phi +\omega +\omega_{1})I, & \\ 0 & = & \phi I-\omega R, & \\ 0 & = & \sigma I+\nu \beta_{e}BS-\omega_{2}B. & \end{array}$$

After simplification, we get


$$\begin{array}{c} F_{0}=(S^{0},E^{0},I^{0},R^{0},B^{0})=(\frac{\propto }{\omega },\;0,\;\;0,\;\;0,\;0), \end{array}$$ (9)

and


$$\begin{array}{c} F^{*}=(S^{*},\;E^{*},\;I^{*},\;R^{*},\;B^{*}), \end{array}$$ (10)

where


$$\begin{array}{rcll} S^{*} & = & \frac{\alpha +\omega }{\beta_{h}\mu }, & \\ E^{*} & = & \frac{\left(\omega +\omega_{1}+\phi \right)\left(\omega (\alpha +\omega )-\beta_{h}\propto \mu \right)\left(\beta_{e}\nu (\alpha +\omega )-\beta_{h}\mu \omega_{2}\right)}{\beta_{h}\mu (\alpha +\omega )\left(\beta_{h}\mu \omega_{2}\left(\omega +\omega_{1}+\phi \right)-\beta_{e}\nu \left(\omega_{1}(\alpha +\omega )+\omega (\alpha +\omega +\phi )\right)\right)}, & \\ I^{*} & = & \frac{\alpha \left(\omega (\alpha +\omega )-\beta_{h}\propto \mu \right)\left(\beta_{e}\nu (\alpha +\omega )-\beta_{h}\mu \omega_{2}\right)}{\beta_{h}\mu (\alpha +\omega )\left(\beta_{h}\mu \omega_{2}\left(\omega +\omega_{1}+\phi \right)-\beta_{e}\nu \left(\omega_{1}(\alpha +\omega )+\omega (\alpha +\omega +\phi )\right)\right)} & \\ R^{*} & = & \frac{\alpha \phi \left(\omega (\alpha +\omega )-\beta_{h}\propto \mu \right)\left(\beta_{e}\nu (\alpha +\omega )-\beta_{h}\mu \omega_{2}\right)}{\beta_{h}\mu \omega (\alpha +\omega )\left(\beta_{h}\mu \omega_{2}\left(\omega +\omega_{1}+\phi \right)-\beta_{e}\nu \left(\omega_{1}(\alpha +\omega )+\omega (\alpha +\omega +\phi )\right)\right)}, & \\ B^{*} & = & \frac{\alpha \phi \left(\omega (\alpha +\omega )-\beta_{h}\propto \mu \right)}{(\alpha +\omega )\left(\beta_{e}\nu \left(\omega_{1}(\alpha +\omega )+\omega (\alpha +\omega +\phi )\right)-\beta_{h}\mu \omega_{2}\left(\omega +\omega_{1}+\phi \right)\right)}. & \end{array}$$

**Theorem 2:** The disease-free equilibrium of the model (2) is locally asymptotically stable if $R_{0}<1$.

**Proof:** That the Jacobian Matrix model is given below.


$$\begin{array}{ccc} J_{\left[SEIRB\right]}(S,E,I,R,B)=\left[\begin{array}{ccccc} w_{1} & -\mu \beta_{h}S & 0 & 0 & -\nu \beta_{e}S\\ \beta_{h}\mu E & w_{2} & 0 & 0 & 0\\ 0 & \alpha & -(\phi +\omega +\omega_{1}) & \;0 & \;0\\ \;0 & \;0 & \phi & \;-\omega & 0\\ \beta_{e}\nu B & \;0 & \;\sigma & 0 & \;\beta_{e}\nu S-\omega_{2} \end{array}\right]. & & \end{array}$$ (11)

Where


$$\begin{array}{cccc} & w_{1}=-\mu \beta_{h}E-\nu \beta_{e}B-\omega & & \\ & w_{2}=-(\omega +\alpha )+\mu \beta_{h}S & & \\ & J_{\left[SEIRB\right]}(\frac{\propto }{\;\omega },\;0,\;0,\;0,\;0)=\left[\begin{array}{ccccc} -\omega & -\frac{\mu \beta_{h}\propto }{\omega } & 0 & -\frac{\nu \beta_{e}\propto }{\omega } & 0\\ \;0 & \frac{\mu \beta_{h}\propto }{\omega }-(\omega +\alpha ) & \;0 & \;0 & \;0\\ \;0 & \alpha & w_{3} & 0 & 0\\ \;0 & \;0 & \phi & \;-\omega & 0\\ \;0 & \;0 & \sigma & 0 & \frac{\nu \beta_{e}\propto }{\omega }-\omega_{2} \end{array}\right]. & \end{array}$$ (12)

Where


$$w_{3}=-\phi -\omega -\omega_{1}$$

So that the given below equation is Characteristic polynomial of (12)


$$\begin{array}{rcll} & & -\left((-\lambda -\omega )(\lambda +\omega )\left(-\lambda -\omega_{2}\right)\left(-\lambda -\omega -\omega_{1}-\phi \right)\left(-\alpha +\frac{\propto \mu \beta_{h}}{\omega }-\lambda -\omega \right)\right)=0. & \end{array}$$

The above model Eigen Values are given below


$$\begin{array}{rcll} \lambda_{1} & = & -\omega , & \\ \lambda_{2} & = & -\omega , & \\ \lambda_{3} & = & \frac{-\alpha \omega +\beta_{h}\propto \mu -\omega^{2}}{\omega }, & \\ \lambda_{4} & = & -\omega -\phi -\omega_{1}, & \\ \lambda_{5} & = & -\omega_{2}. & \end{array}$$

The fact that all of the eigenvalues have negative real values indicates that the system (2) is locally asymptotically stable.

### 3.4 Reproduction number of the generalized Cholera model

The matrices *P* and *Q* are the Jacobian matrices corresponding to the functions *P* and *Q*, respectively, and are examined at the disease-free equilibrium point of $F_{0}$. In the setting of these matrices, the element at the  ( *i* , *j* )  location of matrix *P* denotes the rate at which an infected person in segment *j* transmits the virus to segment *i*. The point at position  ( *i* , *j* )  in the matrix *Q* indicates the propagation of an infection that presently occurs. The reproduction number can be calculated by measuring the $PQ^{-1}$ matrix’s spectrum radius at the disease-free equilibrium state, similarly in [41]. This matrix is known as the “next generation of the matrix,” and it is described as follows:


$$\begin{array}{rcll} J_{0} & = & \left(\begin{array}{ccccc} -\omega & -\frac{\mu \beta_{h}\propto }{\omega } & 0 & 0 & -\frac{\nu \beta_{e}\propto }{\omega }\\ \;0 & \frac{\mu \beta_{h}\propto }{\omega }-(\omega +\alpha ) & \;0 & \;0 & \;0\\ \;0 & \alpha & -\phi -\omega -\omega_{1} & 0 & 0\\ \;0 & \;0 & \phi & \;-\omega & 0\\ \;0 & \;0 & \sigma & 0 & \frac{\nu \beta_{e}\propto }{\omega }-\omega_{2} \end{array}\right) & \\ & & J_{0}=P-Q & \end{array}$$

The following equation can be used to find the vectors *P* and *Q* in our constructed model: 12


$$\begin{array}{rcll} P=\left(\begin{array}{ccccc} 0 & 0 & 0 & 0 & 0\\ 0 & \frac{\propto \mu \beta_{h}}{\omega } & 0 & 0 & 0\\ 0 & \alpha & 0 & 0 & 0\\ 0 & 0 & \phi & 0 & 0\\ 0 & 0 & \sigma & 0 & \frac{\propto \nu \beta_{e}}{\omega } \end{array}\right) & & & \end{array}$$

$$\begin{array}{rcll} Q=\left(\begin{array}{ccccc} \omega & \frac{\propto \mu \beta_{h}}{\omega } & 0 & 0 & \frac{\propto \nu \beta_{e}}{\omega }\\ 0 & \alpha +\omega & 0 & 0 & 0\\ 0 & 0 & \omega +\omega_{1}+\phi & 0 & 0\\ 0 & 0 & 0 & \omega & 0\\ 0 & 0 & 0 & 0 & \omega_{2} \end{array}\right) & & & \end{array}$$

$$\begin{array}{rcll} Q^{-1}=\left(\begin{array}{ccccc} \frac{\alpha +\omega }{\alpha \omega +\omega^{2}} & -\frac{\propto \mu \beta_{h}}{\omega \left(\alpha \omega +\omega^{2}\right)} & 0 & 0 & \frac{-\frac{\alpha \propto \nu \beta_{e}}{\omega }-\propto \nu \beta_{e}}{\omega_{2}\left(\alpha \omega +\omega^{2}\right)}\\ 0 & \frac{\omega_{2}\omega^{3}+\omega_{1}\omega_{2}\omega^{2}+\omega_{2}\omega^{2}\phi }{\omega \omega_{2}\left(\alpha \omega +\omega^{2}\right)\left(\omega +\omega_{1}+\phi \right)} & 0 & 0 & 0\\ 0 & 0 & w_{4} & 0 & 0\\ 0 & 0 & 0 & \frac{1}{\omega } & 0\\ 0 & 0 & 0 & 0 & \frac{1}{\omega_{2}} \end{array}\right) & & & \end{array}$$

Where

$w_{4}=\frac{\alpha \omega_{2}\omega^{2}+\omega_{2}\omega^{3}}{\omega \omega_{2}\left(\alpha \omega +\omega^{2}\right)\left(\omega +\omega_{1}+\phi \right)}$


$$K=P.Q^{-1}$$

So


$$\begin{array}{rcll} K=\left(\begin{array}{ccccc} 0 & 0 & 0 & 0 & 0\\ 0 & \frac{\propto \mu \beta_{h}\left(\omega_{2}\omega^{3}+\omega_{1}\omega_{2}\omega^{2}+\omega_{2}\omega^{2}\phi \right)}{\omega^{2}\omega_{2}\left(\alpha \omega +\omega^{2}\right)\left(\omega +\omega_{1}+\phi \right)} & 0 & 0 & 0\\ 0 & \frac{\alpha \left(\omega_{2}\omega^{3}+\omega_{1}\omega_{2}\omega^{2}+\omega_{2}\omega^{2}\phi \right)}{\omega \omega_{2}\left(\alpha \omega +\omega^{2}\right)\left(\omega +\omega_{1}+\phi \right)} & 0 & 0 & 0\\ 0 & 0 & \frac{\phi \left(\alpha \omega_{2}\omega^{2}+\omega_{2}\omega^{3}\right)}{\omega \omega_{2}\left(\alpha \omega +\omega^{2}\right)\left(\omega +\omega_{1}+\phi \right)} & 0 & 0\\ 0 & 0 & \frac{\sigma \left(\alpha \omega_{2}\omega^{2}+\omega_{2}\omega^{3}\right)}{\omega \omega_{2}\left(\alpha \omega +\omega^{2}\right)\left(\omega +\omega_{1}+\phi \right)} & 0 & \frac{\propto \nu \beta_{e}}{\omega \omega_{2}} \end{array}\right) & & & \end{array}$$

Thus


|K−mI|=0

$$\begin{array}{rcll} \left|\begin{array}{ccccc} -m & 0 & 0 & 0 & 0\\ 0 & \frac{\propto \mu \beta_{h}\left(\omega_{2}\omega^{3}+\omega_{1}\omega_{2}\omega^{2}+\omega_{2}\omega^{2}\phi \right)}{\omega^{2}\omega_{2}\left(\alpha \omega +\omega^{2}\right)\left(\omega +\omega_{1}+\phi \right)}-m & 0 & 0 & 0\\ 0 & \frac{\alpha \left(\omega_{2}\omega^{3}+\omega_{1}\omega_{2}\omega^{2}+\omega_{2}\omega^{2}\phi \right)}{\omega \omega_{2}\left(\alpha \omega +\omega^{2}\right)\left(\omega +\omega_{1}+\phi \right)} & -m & 0 & 0\\ 0 & 0 & \frac{\phi \left(\alpha \omega_{2}\omega^{2}+\omega_{2}\omega^{3}\right)}{\omega \omega_{2}\left(\alpha \omega +\omega^{2}\right)\left(\omega +\omega_{1}+\phi \right)} & -m & 0\\ 0 & 0 & \frac{\sigma \left(\alpha \omega_{2}\omega^{2}+\omega_{2}\omega^{3}\right)}{\omega \omega_{2}\left(\alpha \omega +\omega^{2}\right)\left(\omega +\omega_{1}+\phi \right)} & 0 & \frac{\propto \nu \beta_{e}}{\omega \omega_{2}}-m \end{array}\right|=0 & & & \end{array}$$

By solving the determinant of the mentioned above matrix, we obtain the values of *m*.


$$\begin{array}{rcll} m_{1} & = & 0, & \\ m_{2} & = & 0, & \\ m_{3} & = & 0, & \\ m_{4} & = & \frac{\propto \mu \beta_{h}}{\omega (\alpha +\omega )}, & \\ m_{5} & = & \frac{\propto \nu \beta_{e}}{\omega \omega_{2}}. & \end{array}$$

Considering that the reproductive number $R_{0}$ and the primary eigenvalue of the matrix $PQ^{-1}$ are associated as follows:


$$R_{0}=\frac{\propto \nu \beta_{e}}{\omega \omega_{2}}.$$

### 3.5 Sensitivity analysis

Sensitivity analysis is helpful in figuring out how various parameters, particularly those dealing with unclear data, affect a model’s stability in relation to one another.

Additionally, this study aids in determining the most crucial characteristics. Since the number of reproductions is.


$$R_{0}=\frac{\propto \nu \beta_{e}}{\omega \omega_{2}}.$$

By calculating the partial derivatives of the criterion with respect to the pertinent parameters, we can investigate the sensitivity of $R_{0}$ in the following ways:

It is clear that when we adjust the settings, the value of $R_{0}$ is quite sensitive. The parameters $\beta_{h}$,  ∝ , and *μ* show expansion in our analysis, whereas *α* and *ω* show contraction. Therefore, medication shouldn’t occur before treatment for effective elimination of infections.

$$\begin{array}{rcll} \frac{\partial R_{0}}{\partial \propto } & = & \frac{\nu \beta_{e}}{\omega \omega_{2}}>0, & \\ \frac{\partial R_{0}}{\partial \nu } & = & \frac{\propto \beta_{e}}{\omega \omega_{2}}>0, & \\ \frac{\partial R_{0}}{\partial \beta_{e}} & = & \frac{\propto \nu }{\omega \omega_{2}}>0, & \\ \frac{\partial R_{0}}{\partial \omega } & = & -\frac{\propto \nu \beta_{e}}{\omega^{2}\omega_{2}}<0, & \\ \frac{\partial R_{0}}{\partial \alpha } & = & -\frac{\propto \nu \beta_{e}}{\omega \omega_{2}^{2}}<0. & \end{array}$$

**Fig 2:**
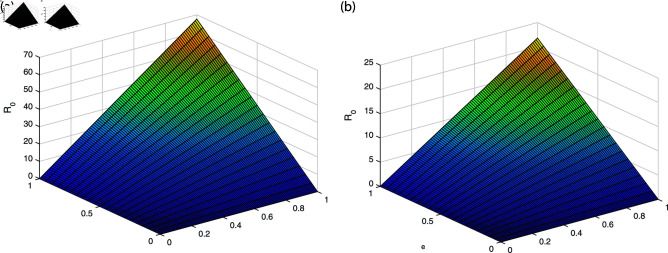
The behavior of *ν* and $\beta_{\text{e}}$ with respect to  ∝  (a) *ν* with respect to parameter  ∝  (b) $\beta_{\text{e}}$ with respect to parameter  ∝ .

**Fig 3:**
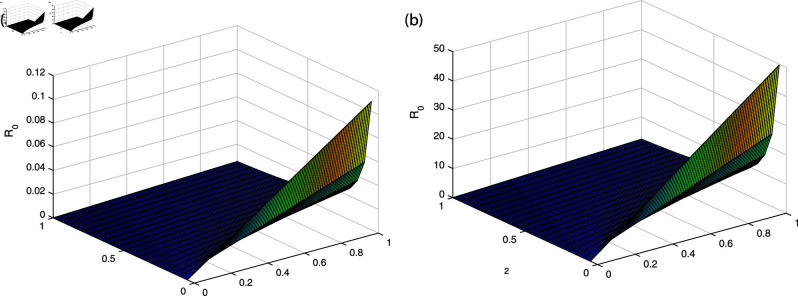
The behavior of *ω* and $\omega_{2}$ with respect to  ∝  (a) *ω* with respect to parameter  ∝  (b) $\omega_{2}$ with respect to parameter  ∝ .

**Fig 4:**
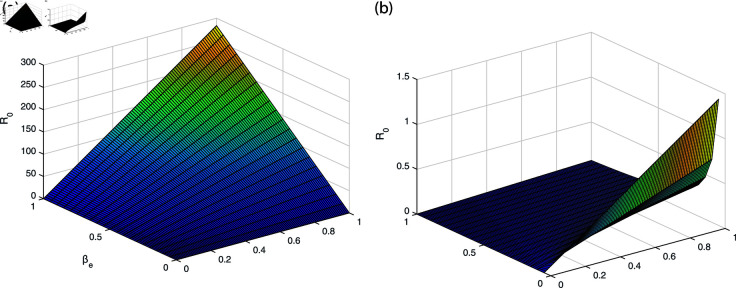
The behavior of $\beta_{\text{e}}$ and *ω* with respect to *ν* (a) $\beta_{\text{e}}$ with respect to parameter *ν* (b) *ω* with respect to parameter *ν.*

**Fig 5:**
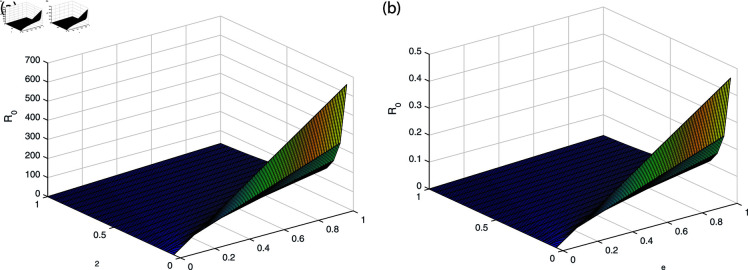
The behavior of $\omega_{2}$ and *ω* with respect to *ν* and $\beta_{\text{e}}$ (a) $\omega_{2}$ with respect to parameter *ν* (b) *ω* with respect to parameter $\beta_{\text{e}}$.

**Fig 6:**
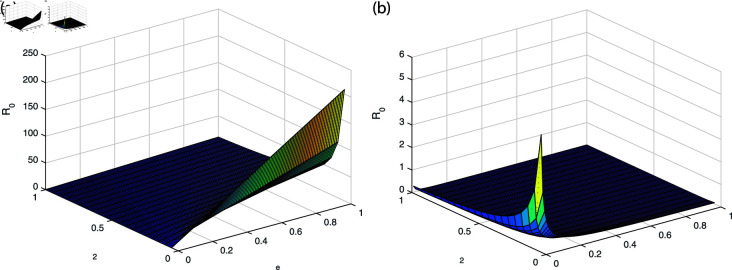
The behavior of $\omega_{2}$ and *ω* with respect to $\beta_{\text{e}}$ and $\omega_{2}$ (a) $\omega_{2}$ with respect to parameter $\beta_{\text{e}}$ (b) *ω* with respect to parameter $\omega_{2}$.

It can be observed in Figs 2, 3, 4, 5, and 6 that the behavior of the rate of change of $R_{0}$ with respect to different parameters impact which suggests that it is highly responsive. The behavior of *ν* and $\beta_{e}$ with respect to  ∝  and the behavior of $\beta_{e}$ with respect to *ν* is approximately similar with only minor variations. Similarly, the behavior of *ω* with respect to  ∝ ,  *ν* ,  and $\beta_{e}$ and the behavior of $\omega_{2}$ with respect to $\beta_{e}$ and *ω* shows analogous patterns, with minor effects observed. All the sub-figures indicate that the rate of change of each parameter provide bounded results and is in specified range, which is important for maintaining stable conditions.

## 4 Impact of global derivative

Riemann-Stieltjes integration is a commonly recognized integration method that is often used to get the integral of a function over its curve. If


H(y)= ∫ h(y)dy.

The given function *h*(*y*) with respect to *g*(*y*) has the riemann-stieltijes integral


$$H_{g}(y)=\int h(y)dg(y).$$

The definition of the global derivative, which has a close connection to this integral, is given as follows


Dgf(y)= lim ⁡ ℏ→0f(y+ℏ)−f(y)g(y+ℏ)−g(y).

By the definition of the derivative, we get


$$D_{g}f(y)=\frac{\overset{´}{f}(y)}{ǵ(y)},$$

where $ǵ(y)\neq 0,\text{for}\text{all}y\in D_{ǵ}$


$$\begin{array}{cccc} D_{g}S(t)= & \propto -\mu \beta_{h}SE-\nu \beta_{e}SB-\omega S, & & \\ D_{g}E(t)= & \mu \beta_{h}SE-\alpha E-\omega E, & & \\ D_{g}I(t)= & \alpha E-\phi I-\omega I, & & \\ D_{g}B(t)= & \sigma I+\nu \beta_{e}SB-\omega B, & & \\ D_{g}R(t)= & \phi I-\omega R. & \end{array}$$ (13)

For the sake of simplicity, *g* will be taken to be different. Thus


$$\begin{array}{c} \begin{array}{ccc} Ś= & ǵ[\propto -\mu \beta_{h}SE-\nu \beta_{e}SB-\omega S]=M_{1}[t,\eta ], & \\ É= & ǵ[\mu \beta_{h}SE-\alpha E-\omega E]=M_{2}[t,\eta ],\\ Í= & ǵ[\alpha E-\phi I-\omega I]=M_{3}[t,\eta ],\\ \overset{´}{B}= & ǵ[\sigma I+\nu \beta_{e}SB-\omega B]=M_{4}[t,\eta ],\\ Ŕ= & ǵ[\phi I-\omega R]=M_{5}[t,\eta ], \end{array} \end{array}$$ (14)

where


η=S,E,I,B,R

∥ǵ∥∞= sup ⁡ n∈Dg′<N,

The following condition $M(t,S,I,E,B,R)<k\left(1+|S{|}^{2}\right)\forall \;S_{1}\;\text{and}\;S_{2}$ We have $∥M(t,S_{1},E,I,B,R)-M(t,S_{2},E,I,B,R)∥<K∥S_{1}-S_{2}{∥}_{\infty }^{2}.$

Initially


$$\begin{array}{llll} & |M_{1}(t,S_{1},E,I,B,R){|}^{2}=|ǵ[\propto -\mu \beta_{h}S_{1}E-\nu \beta_{e}S_{1}B-\omega S_{1}]{|}^{2},\; & & \;\\ & |M_{1}(t,S_{1},E,I,B,R){|}^{2}=|ǵ{|}^{2}|[\propto -(\mu \beta_{h}E+\nu \beta_{e}B+\omega )S_{1}]{|}^{2},\; & & \;\\ & |M_{1}(t,S_{1},E,I,B,R){|}^{2}\leq 2|ǵ{|}^{2}[|\propto {|}^{2}+{\left(\mu \beta_{h}E+\nu \beta_{e}B+\omega \right)}^{2}|S_{1}{|}^{2}],\; & & \;\\ & |M_{1}(t,S_{1},E,I,B,R){|}^{2}\leq 2|ǵ{|}^{2}[|\propto {|}^{2}+\{2{\left(\mu \beta_{h}\right)}^{2}|E{|}^{2}+4{\left(\nu \beta_{e}\right)}^{2}|B{|}^{2}+4{\left(\omega \right)}^{2}\}|S_{1}{|}^{2}],\; & & \;\\ & |M_{1}(t,S_{1},E,I,B,R){|}^{2}\leq 2|ǵ{|}^{2}|\propto {|}^{2}\left[1+\frac{\{2{\left(\mu \beta_{h}\right)}^{2}|E{|}^{2}+4{\left(\nu \beta_{e}\right)}^{2}|B{|}^{2}+4{\left(\omega \right)}^{2}\}|S_{1}{|}^{2}}{\propto^{2}}\right],\; & & \;\\ & |M_{1}(t,S_{1},E,I,B,R){|}^{2}<K_{1}(1+|S_{1}{|}^{2}),\; & & \; \end{array}$$

under the condition


$$\frac{\{2{\left(\mu \beta_{h}\right)}^{2}|E{|}^{2}+4{\left(\nu \beta_{e}\right)}^{2}|B{|}^{2}+4{\left(\omega \right)}^{2}\}|S_{1}{|}^{2}}{\propto^{2}}<1,$$

where


$$\begin{array}{llll} & K_{1}=2|ǵ{|}^{2}|\propto {|}^{2}.\; & & \;\\ & |M_{2}(t,S,E_{1},I,B,R){|}^{2}=|ǵ[\mu \beta_{h}SE_{1}-\alpha E_{1}-\omega E_{1}]{|}^{2},\; & & \;\\ & |M_{2}(t,S,E_{1},I,B,R){|}^{2}=|ǵ{\left[\mu \beta_{h}S-(\alpha +\omega )\right]}^{2}|E_{1}{|}^{2},\; & & \;\\ & |M_{2}(t,S,E_{1},I,B,R){|}^{2}\leq 2|ǵ{|}^{2}|[\mu^{2}\beta_{h}^{2}|S{|}^{2}+{\left(\alpha +\omega \right)}^{2}]|E_{1}{|}^{2},\; & & \;\\ & |M_{2}(t,S,E_{1},I,B,R){|}^{2}\leq 2|ǵ{|}^{2}|[\mu^{2}\beta_{h}^{2}|S{|}^{2}+2\alpha^{2}+2\omega^{2}]|E_{1}{|}^{2},\; & & \;\\ & |M_{2}(t,S,E_{1},I,B,R){|}^{2}\leq 2|ǵ{|}^{2}(2\alpha^{2}+2\omega^{2})\left[1+\frac{\mu^{2}\beta_{h}^{2}|S{|}^{2}}{2\alpha^{2}+2\omega^{2}}\right]|E_{1}{|}^{2},\; & & \;\\ & |M_{2}(t,S,E_{1},I,B,R){|}^{2}<K_{2}[1+|S{|}^{2}],\; & & \; \end{array}$$

under the condition


$$\frac{\mu^{2}\beta_{h}^{2}|S{|}^{2}}{2\alpha^{2}+2\omega^{2}}<1,$$

where


$$\begin{array}{llll} & K_{2}=2|ǵ{|}^{2}(2\alpha^{2}+2\omega^{2})|E{|}^{2}.\; & & \;\\ & |M_{3}(t,S,E,I_{1},B,R){|}^{2}=|ǵ[\alpha E-\phi I_{1}-\omega_{1}I_{1}-\omega I_{1}]{|}^{2},\; & & \;\\ & |M_{3}(t,S,E,I_{1},B,R){|}^{2}=|ǵ{|}^{2}|[\alpha E-(\phi +\omega_{1}+\omega )I_{1}]{|}^{2},\; & & \;\\ & |M_{3}(t,S,E,I_{1},B,R){|}^{2}\leq 2|ǵ{|}^{2}[|\alpha E{|}^{2}+{\left(\phi +\omega_{1}+\omega \right)}^{2}|I_{1}{|}^{2}],\; & & \;\\ & |M_{3}(t,S,E,I_{1},B,R){|}^{2}\leq 2|ǵ{|}^{2}[\alpha^{2}|E{|}^{2}+\{2\phi^{2}+2\omega_{1}^{2}+2\omega^{2}\}|I_{1}{|}^{2}],\; & & \;\\ & |M_{3}(t,S,E,I_{1},B,R){|}^{2}\leq 2|ǵ{|}^{2}\alpha^{2}|E{|}^{2}\left[1+\frac{\{2\phi^{2}+2\omega_{1}^{2}+2\omega^{2}\}|I_{1}{|}^{2}}{\alpha^{2}|E{|}^{2}}\right],\; & & \;\\ & |M_{3}(t,S,E,I_{1},B,R){|}^{2}<K_{3}[1+|I_{1}{|}^{2}],\; & & \; \end{array}$$

under the condition


$$\frac{\left|I_{1}{|}^{2}\{2\phi^{2}+2\omega_{1}^{2}+2\omega^{2}\right\}}{|E{|}^{2}\alpha^{2}}<1,$$

where


$$\begin{array}{llll} & K_{3}=2|ǵ{|}^{2}\alpha^{2}|E_{1}{|}^{2}.\; & & \;\\ & |M_{4}(t,R_{1}){|}^{2}=|ǵ[\phi I-\omega R_{1}]{|}^{2},\; & & \;\\ & |M_{4}(t,R_{1}){|}^{2}=[|ǵ{|}^{2}]|[\phi I-\omega R_{1}]{|}^{2},\; & & \;\\ & |M_{4}(t,R_{1}){|}^{2}\leq 2[|ǵ{|}^{2}][\phi^{2}|I{|}^{2}+\omega^{2}|R_{1}{|}^{2}],\; & & \;\\ & |M_{4}(t,R_{1}){|}^{2}\leq 2|ǵ{|}^{2}\phi^{2}|I{|}^{2}\left[1+\frac{\omega^{2}|R_{1}{|}^{2}}{\phi^{2}|I{|}^{2}}\right],\; & & \;\\ & |M_{4}(t,,R_{1}){|}^{2}<K_{5}\left[1+|R_{1}{|}^{2}\right],\; & & \; \end{array}$$

under the situation


$$\frac{\omega^{2}|R_{1}{|}^{2}}{\phi^{2}|I{|}^{2}}<1,$$

where


$$\begin{array}{llll} & K_{4}=2|ǵ{|}^{2}\phi^{2}|I{|}^{2}.\; & & \;\\ & |M_{5}(t,B_{1}){|}^{2}=|ǵ[\sigma I+\nu \beta_{e}SB_{1}-\omega_{2}B_{1}]{|}^{2},\; & & \;\\ & |M_{5}(t,B_{1}){|}^{2}={\left(ǵ\right)}^{2}|[\sigma I+(\nu \beta_{e}S-\omega_{2})B_{1}]{|}^{2},\; & & \;\\ & |M_{5}(t,B_{1}){|}^{2}\leq 2{\left(ǵ\right)}^{2}[\sigma^{2}|I{|}^{2}+{\left(\nu \beta_{e}S-\omega_{2}\right)}^{2}|B_{1}{|}^{2}],\; & & \;\\ & |M_{5}(t,B_{1}){|}^{2}\leq 2{\left(ǵ\right)}^{2}[\sigma^{2}|I{|}^{2}+\{2\nu^{2}\beta_{e}^{2}|S{|}^{2}+2\omega_{2}^{2}\}|B_{1}{|}^{2}],\; & & \;\\ & |M_{5}(t,s,E,I,B_{1},R){|}^{2}\leq 2\sigma^{2}{\left(ǵ\right)}^{2}|I{|}^{2}\left[1+\frac{\{2\nu^{2}\beta_{e}^{2}|S{|}^{2}+2\omega_{2}^{2}\}|B_{1}{|}^{2}}{\sigma^{2}|I{|}^{2}}\right],\; & & \;\\ & |M_{5}(t,B_{1}){|}^{2}<K_{4}[1+|B_{1}{|}^{2}],\; & & \; \end{array}$$

under the situation


$$\frac{\{2\nu^{2}\beta_{e}^{2}|S{|}^{2}+2\omega_{2}^{2}\}|B_{1}{|}^{2}}{\sigma^{2}|I{|}^{2}}<1,$$

where


$$K_{5}=2{\left(ǵ\right)}^{2}\sigma^{2}{\left(|I|\right)}^{2}.$$

Hence the linear growth criteria is satisfied. Now we verify the Lipschitz condition as follows. If


|M1(t,s1)−M1(t,S2)|2=|[−βhμE−νβeB−ω]|2|(S1−S2)|2,|M1(t,s1)−M1(t,S2)|2=|[−(βhμE+νβeB)−ω]|2|(S1−S2)|2,|M1(t,s1)−M1(t,S2)|2≤[2(βhμE+νβeB)2+2(ω)2]|(S1−S2)|2,|M1(t,s1)−M1(t,S2)|2≤[4μ2βh2|E|2+4ν2βe2|B|2+2ω2]|(S1−S2)|2,|M1(t,s1)−M1(t,S2)|2≤[4μ2βh2 sup ⁡ t∈DE|E|2+4ν2βe2 sup ⁡ t∈DB|B|2+2ω2]sup ⁡ t∈D(S1−S2)|(S1−S2)|2,|M1(t,s1)−M1(t,S2)|2≤[4μ2βh2∥E∥∞2+4ν2βe2∥B∥∞2+2ω2]∥(S1−S2)∥∞2,|M1(t,s1)−M1(t,S2)|2≤K¯1∥(S1−S2)∥∞2,

where


K¯1=4μ2βh2∥E∥∞2+4ν2βe2∥B∥∞2+2ω2.|M2(t,E1)−M2(t,E2)|2=|[μβhS−α−ω]|2|(E1−E2)|2,|M2(t,E1)−M2(t,E2,I,B,R)|2≤[2|μβhS|2+2|α+ω|2]|(E1−E2)|2,|M2(t,E1)−M2(t,E2)|2≤[2|μ2βh2|S|2+4α2+4ω2]|(E1−E2)|2,|M2(t,E1)−M2(t,E2)|2≤[2|μ2βh2 sup ⁡ t∈DS|S|2+4α2+4ω2]sup ⁡ t∈D(E1−E2)|(E1−E2)|2,|M2(t,E1)−M2(t,E2)|2≤[2|μ2βh2∥S∥∞2+4α2+4ω2]∥(E1−E2)∥∞2,|M2(t,E1)−M2(t,E2)|2≤K2¯∥(E1−E2)∥∞2,

where


K¯2=2|μ2βh2∥S∥∞2+4α2+4ω2.|M3(t,I1)−M3(t,I2)|2=|[−ϕ−ω1−ω]|2|(I1−I2)|2,|M3(t,I1)−M3(t,I2)|2≤[2ϕ2+2ω12+2ω2]sup ⁡ t∈D(I1−I2)|(I1−I2)|2,|M3(t,I1)−M3(t,I2)|2≤[2ϕ2+2ω12+2ω2]∥(I1−I2)∥∞2,|M3(t,I1)−M3(t,I2)|2≤K3¯∥(I1−I2)∥∞2,

where


K¯3=2ϕ2+2ω12+2ω2.|M4(t,R1)−M4(t,R2)|2=|ω|2|R1−R2|2,|M4(t,R1)−M4(t,R2)|2=|ω|2|R1−R2|2,|M4(t,R1)−M4(t,R2)|2=ω2|R1−R2|2,|M4(t,R1)−M4(t,R2)|2≤ω2 sup ⁡ t∈D(R1−R2)|R1−R2|2,|M4(t,R1)−M4(t,R2)|2≤ω2∥(R1−R2)∥∞2,|M4(t,R1)−M4(t,R2)|2≤K5¯∥(R1−R2)∥∞2,

where


$$\bar{K_{4}}=\omega^{2}.$$

|M5(t,B1)−M5(t,B2)|2=|νβeS−ω2|2|(B1−B2)|2,|M5(t,B1)−M5(t,B2)|2=[2ν2βe2|S|2+2ω22]|(B1−B2)|2,|M5(t,B1)−M5(t,B2)|2=[2ν2βe2 sup ⁡ t∈D(s)|S|2+2ω22]sup ⁡ t∈D(B1−B2)|(B1−B2)|2,|M5(t,B1)−M5(t,B2)|2=[2ν2βe2∥S∥∞2+2ω22]∥(B1−B2)∥∞2,|M5(t,B1)−M5(t,B2)|2≤K4¯∥(B1−B2)∥∞2,

where


$$\bar{K_{5}}=2\nu^{2}\beta_{e}^{2}∥S{∥}_{\infty }^{2}+2\omega_{2}^{2}.$$

The system 2 therefore has a unique solution under the following conditions using global derivative impact results.


$$\begin{array}{cccc} max\{\begin{array}{cc} \frac{\{2{\left(\mu \beta_{h}\right)}^{2}|E{|}^{2}+4{\left(\nu \beta_{e}\right)}^{2}|B{|}^{2}+4{\left(\omega \right)}^{2}\}|S_{1}{|}^{2}}{\propto^{2}}, & \\ \frac{\mu^{2}\beta_{h}^{2}|S{|}^{2}}{2\alpha^{2}+2\omega^{2}}, & \\ \frac{\left|I_{1}{|}^{2}\{2\phi^{2}+2\omega_{1}^{2}+2\omega^{2}\right\}}{|E{|}^{2}\alpha^{2}}, & \\ \frac{\omega^{2}|R_{1}{|}^{2}}{\phi^{2}|I{|}^{2}}, & \\ \frac{\{2\nu^{2}\beta_{e}^{2}|S{|}^{2}+2\omega_{2}^{2}\}|B_{1}{|}^{2}}{\sigma^{2}|I{|}^{2}}, & \end{array} & < & 1 & \end{array}$$

## 5 Global stability analysis

The Lyapunovs approach and LaSalles invariance concept are used to show the global stability assessment and estimate the necessary conditions for the eradication of disease see in [42].

### 5.1 Lyapunov first derivative

The Lyapunov function for the endemic equilibrium, denoted as $\left\{S^{*},E^{*},I^{*},R^{*},B^{*}\right\}$, with *L* > 0, is represented by $F^{*}$.

**Theorem 3:** If the reproductive number $R_{0}>1$ , then the endemic *F* ∗  of the cholera disease outbreak is globally asymptotically stable.

**Proof:** By definition, we get


L(S∗,E∗,I∗,B∗,R∗)= {S−S∗−S∗log ⁡ S∗S}+ {E−E∗−E∗log ⁡ E∗E}+ {I−I∗−I∗log ⁡ I∗I}+ {B−B∗−B∗log ⁡ B∗B}+ {R−R∗−R∗log ⁡ R∗R}dLdt=(S−S∗S)ddtS+(E−E∗E)ddtE+(I−I∗I)ddtI+(B−B∗B)ddtB+(R−R∗R)ddtR=(S−S∗S) {∝−μβhSE−νβeSB−ωS }+(E−E∗E) {μβhSE−αE−ωE }+(I−I∗I) {αE−ϕI−ω1I−ωI }+(R−R∗R) {ϕI−ωR }+(B−B∗B) {σI+νβeSB−ω2B }

Substituting $S=(S-S^{*})$, $\;E=(E-\;E^{*})$, $\;I=(I-\;I^{*})$, $\;B=(B-\;B^{*})$, and $\;R=(R-\;R^{*})$.


$$\begin{array}{rcll} \frac{dL}{dt} & = & (\frac{S-S^{*}}{S})\{\propto -(\mu \beta_{h}(E-E^{*})+\nu \beta_{e}(B-B^{*})+\omega )(S-S^{*})\} & \\ & & +(\frac{E-E^{*}}{E})\{(\mu \beta_{h}(S-S^{*})-\alpha -\omega )(E-E^{*})\} & \\ & & +(\frac{I-I^{*}}{I})\{\alpha (-E^{*}+E)-(\phi +\omega_{1}+\omega )(-I^{*}+I)\} & \\ & & +(\frac{B-B^{*}}{B})\{\sigma (I-I^{*})+(\nu \beta_{e}(S-S^{*})-\omega_{2})(B-B^{*})\} & \\ & & +(\frac{R-R^{*}}{R})\{\phi (-I^{*}+I)-\omega (R-R^{*})\}, & \\ \frac{dL}{dt} & = & \propto +\frac{1}{S}\left(\propto S^{*}\right)-\frac{1}{S}\left(\mu \beta_{h}{\left(S-S^{*}\right)}^{2}E\right)+\frac{1}{S}\left(\mu \beta_{h}{\left(S-S^{*}\right)}^{2}E^{*}\right)- & \\ & & \frac{1}{S}\left(\nu \beta_{e}{\left(S-S^{*}\right)}^{2}\right)+\frac{1}{S}\left(\nu \beta_{e}{\left(S-S^{*}\right)}^{2}B^{*}\right)-\frac{1}{S}\left(\omega {\left(S-S^{*}\right)}^{2}\right)+ & \\ & & \frac{1}{E}\left(\mu \beta_{h}{\left(E-E^{*}\right)}^{2}S\right)-\frac{1}{E}\left(\mu \beta_{h}{\left(E-E^{*}\right)}^{2}S^{*}\right)-\frac{1}{E}\left(\alpha {\left(E-E^{*}\right)}^{2}\right) & \\ & & -\frac{1}{E}\left(\omega {\left(E-E^{*}\right)}^{2}\right)+\alpha E-\alpha E^{*}-\frac{1}{I}\left(\alpha EI^{*}\right)-\frac{1}{I}\left(\phi {\left(I-I^{*}\right)}^{2}\right) & \\ & & +\frac{1}{I}\left(\alpha E^{*}I^{*}\right)-\frac{1}{I}\left(\omega_{1}{\left(I-I^{*}\right)}^{2}\right)-\frac{1}{I}\left(\omega {\left(I-I^{*}\right)}^{2}\right)+\sigma I-\sigma I^{*}- & \\ & & \frac{1}{B}\left(\sigma IB^{*}\right)+\frac{\sigma I^{*}B^{*}}{B}+\frac{1}{B}\left(\nu \beta_{e}{\left(B-B^{*}\right)}^{2}S\right)-\frac{1}{B}\left(\nu \beta_{e}{\left(B-B^{*}\right)}^{2}S^{*}\right) & \\ & & -\frac{1}{B}\left(\omega_{2}{\left(B-B^{*}\right)}^{2}\right)+\phi I-\phi I^{*}-\frac{1}{R}\left(\phi IR^{*}\right)+\frac{1}{R}\left(\phi R^{*}I^{*}\right) & \\ & & -\frac{1}{R}\left(\omega {\left(R-R^{*}\right)}^{2}\right), & \end{array}$$

which can be written as


$$\frac{dL}{dt}=\;𝔑-ℜ,$$

where


$$\begin{array}{rcll} 𝔑 & = & \propto +\frac{1}{S}\left(\propto S^{*}\right)+\frac{1}{S}\left(\mu \beta_{h}{\left(S-S^{*}\right)}^{2}E^{*}\right)+\frac{1}{S}\left(\nu \beta_{e}{\left(S-S^{*}\right)}^{2}B^{*}\right) & \\ & & +\frac{1}{E}\left(\mu \beta_{h}{\left(E-E^{*}\right)}^{2}S\right)+\frac{1}{I}\left(\alpha E^{*}I^{*}\right)+\frac{1}{B}\left(\sigma I^{*}B^{*}\right)+ & \\ & & \frac{1}{B}\left(\nu \beta_{e}{\left(B-B^{*}\right)}^{2}S\right)+\alpha E+\sigma I+\phi I+\frac{1}{R}\left(\phi I^{*}R^{*}\right), & \end{array}$$

and


$$\begin{array}{rcll} ℜ & = & \frac{1}{S}\left(\mu \beta_{h}{\left(S-S^{*}\right)}^{2}E\right)+\frac{1}{S}\left(\nu \beta_{e}{\left(S-S^{*}\right)}^{2}\right)+\frac{1}{S}\left(\omega {\left(S-S^{*}\right)}^{2}\right)+ & \\ & & \frac{1}{E}\left(\mu \beta_{h}{\left(E-E^{*}\right)}^{2}S^{*}\right)+\frac{1}{E}\left(\alpha {\left(E-E^{*}\right)}^{2}\right)+\alpha E^{*}+\frac{1}{E}\left(\omega {\left(E-E^{*}\right)}^{2}\right) & \\ & & +\frac{1}{I}\left(\phi {\left(I-I^{*}\right)}^{2}\right)+\frac{1}{I}\left(\omega_{1}{\left(I-I^{*}\right)}^{2}\right)+\frac{1}{I}\left(\omega {\left(I-I^{*}\right)}^{2}\right)+\frac{1}{I}\left(\alpha EI^{*}\right) & \\ & & +\frac{1}{B}\left(\sigma IB^{*}\right)+\frac{1}{B}\left(\nu \beta_{e}{\left(B-B^{*}\right)}^{2}S^{*}\right)+\frac{1}{B}\left(\omega_{2}{\left(B-B^{*}\right)}^{2}\right) & \\ & & +\frac{1}{R}\left(\phi IR^{*}\right)+\frac{1}{R}\left(\omega {\left(R-R^{*}\right)}^{2}\right)+\sigma I^{*}+\phi I^{*}. & \end{array}$$

We conclude that if *F* ∗ , this yields ${}_{0}^{FFM}D_{t}^{\xi ,\tau }L<0$, However, in the case where the $\;S=\;S^{*}$, $\;E=\;E^{*}$, $\;I=\;I^{*}$, $\;B=\;B^{*}$, and $\;R=\;R^{*}$.

$𝔑<ℜ\;\Rightarrow {\;}_{0}^{FFM}D_{t}^{\xi ,\tau }L=0$.

Now

$\left\{(S^{*},E^{*},I^{*},B^{*},R^{*})\in \Gamma :{\;}_{0}^{FFM}D_{t}^{\xi ,\tau }L=0\right\}$* illustrates the point* $F^{*}$ for the accomplished model.

In accordance with Lasalles’ theory of consistency, the system $F^{*}$ *is uniform stability across the globe in Γ if Γ*.

## 6 Computational analysis with Fractal-Fractional operator

The requirements that need to be fulfilled in order for our solution to attain global stability are examined in this part, and we make understanding of them by utilizing to Lyapunov functions and the LaSalle principle. Using the Fractal Fractional with Mittag-Leff1er Kernel Model 1 so we have


$$\begin{array}{rcll} {\;}_{\;0}^{\;FFM}D_{t}^{\xi ,\;\tau }S(t) & =S_{1}(t,\Gamma ), & & \\ {\;}_{\;0}^{\;FFM}D_{t}^{\xi ,\;\tau }E(t) & =E_{1}(t,\Gamma ), & & \\ {\;}_{\;0}^{\;FFM}D_{t}^{\xi ,\;\tau }I(t) & =I_{1}(t,\Gamma ), & & \\ {\;}_{\;0}^{\;FFM}D_{t}^{\xi ,\;\tau }B(t) & =B_{1}(t,\Gamma ), & & \\ {\;}_{\;0}^{\;FFM}D_{t}^{\xi ,\;\tau }R(t) & =R_{1}(t,\Gamma ). & & \end{array}$$

By using Mittag Laff1er kernel, we get


$$\begin{array}{rcll} S(t_{\left(1+\xi \right)}) & = & S_{0}+\frac{(1-\tau )\xi }{AB(\tau )}t_{\xi }^{\tau -1}S_{1}(M(t_{\xi }))+\hbar \overset{\xi }{\underset{ℜ=0}{\sum }}S_{1}(\varrho ,\Gamma )\varrho^{\tau -1}(t_{\xi +1}-\varrho )d\varrho , & \\ E(t_{\left(1+\xi \right)}) & = & E_{0}+\frac{1-\tau }{AB(\tau )}t_{\xi }^{\tau -1}E_{1}(M(t_{\xi }))+\hbar \overset{\xi }{\underset{ℜ=0}{\sum }}E_{1}(\varrho ,\Gamma )\varrho^{\tau -1}(t_{\xi +1}-\varrho )d\varrho , & \\ I(t_{\left(1+\xi \right)}) & = & I_{0}+\frac{1-\tau }{AB(\tau )}t_{\xi }^{\tau -1}I_{1}(M(t_{\xi }))+\hbar \overset{\xi }{\underset{ℜ=0}{\sum }}I_{1}(\varrho ,\Gamma )\varrho^{\tau -1}(t_{\xi +1}-\varrho )d\varrho , & \\ B(t_{\left(1+\xi \right)}) & = & B_{0}+\frac{1-\tau }{AB(\tau )}t_{\xi }^{\tau -1}B_{1}(M(t_{\xi }))+\hbar \overset{\xi }{\underset{ℜ=0}{\sum }}B_{1}(\varrho ,\Gamma )\varrho^{\tau -1}(t_{\xi +1}-\varrho )d\varrho , & \\ R(t_{\left(1+\xi \right)}) & = & R_{0}+\frac{1-\tau }{AB(\tau )}t_{\xi }^{\tau -1}R_{1}(M(t_{\xi }))+\hbar \overset{\xi }{\underset{ℜ=0}{\sum }}R_{1}(\varrho ,\Gamma )\varrho^{\tau -1}(t_{\xi +1}-\varrho )d\varrho . & \end{array}$$

where


$$\begin{array}{rcll} M(t_{\xi }) & = & (t_{\xi },B(t_{\xi }),S(t_{\xi }),E(t_{\xi }),I(t_{\xi }),R(t_{\xi })). & \end{array}$$

Now


$$\begin{array}{rcll} S^{\xi +1} & =S_{0}+\frac{\tau {\left(△t\right)}^{\tau }}{\Gamma (\xi +2)}\overset{\xi }{\underset{ℜ=0}{\sum }}[t_{ℜ}^{\tau -1}S_{1}(M(t_{\xi }))\left\{г\right\} & & \\ & \;-t_{ℜ-1}^{\tau -1}S_{1}(N(t_{\xi +1}))({\left(\xi -ℜ+1\right)}^{\tau +1}-{\left(\xi -ℜ\right)}^{\tau }(\xi -ℜ+1+\tau ))], & & \\ E^{\xi +1} & =E_{0}+\frac{\tau {\left(△t\right)}^{\tau }}{\Gamma (\xi +2)}\overset{\xi }{\underset{ℜ=0}{\sum }}[t_{ℜ}^{\tau -1}E_{1}(M(t_{\xi }))(г) & & \\ & \;-t_{ℜ-1}^{\tau -1}E_{1}(N(t_{\xi +1}))({\left(\xi -ℜ+1\right)}^{\tau +1}-{\left(\xi -ℜ\right)}^{\tau }(\xi -ℜ+1+\tau ))], & & \\ I^{\xi +1} & =I_{0}+\frac{\tau {\left(△t\right)}^{\tau }}{\Gamma (\xi +2)}\overset{\xi }{\underset{ℜ=0}{\sum }}[t_{ℜ}^{\tau -1}I_{1}(M(t_{\xi }))(г) & & \\ & \;-t_{ℜ-1}^{\tau -1}I_{1}(N(t_{\xi +1}))({\left(\xi -ℜ+1\right)}^{\tau +1}-{\left(\xi -ℜ\right)}^{\tau }(\xi -ℜ+1+\tau ))], & & \\ B^{\xi +1} & =B_{0}+\frac{\tau {\left(△t\right)}^{\tau }}{\Gamma (\xi +2)}\overset{\xi }{\underset{ℜ=0}{\sum }}[t_{ℜ}^{\tau -1}B_{1}(M(t_{\xi }))(г) & & \\ & \;-t_{ℜ-1}^{\tau -1}B_{1}(N(t_{\xi +1}))({\left(\xi -ℜ+1\right)}^{\tau +1}-{\left(\xi -ℜ\right)}^{\tau }(\xi -ℜ+1+\tau ))], & & \\ R^{\xi +1} & =R_{0}+\frac{\tau {\left(△t\right)}^{\tau }}{\Gamma (\xi +2)}\overset{\xi }{\underset{ℜ=0}{\sum }}[t_{ℜ}^{\tau -1}R_{1}(M(t_{\xi }))(г) & & \\ & \;-t_{ℜ-1}^{\tau -1}R_{1}(N(t_{\xi +1}))({\left(\xi -ℜ+1\right)}^{\tau +1}-{\left(\xi -ℜ\right)}^{\tau }(\xi -ℜ+1+\tau ))]. & & \end{array}$$

where


$$\begin{array}{rcll} \left(N(t_{\xi +1})\right) & = & (t_{1+\xi },S(t_{\xi +1}),I(t_{\xi +1}),E(t_{\xi }),B(t_{\xi +1}),R(t_{\xi +1})), & \\ г & = & {\left(\xi -ℜ+1\right)}^{\tau }(\xi -ℜ+2+\tau )-{\left(\xi -ℜ\right)}^{\tau }(\xi -ℜ+2+2\tau ). & \end{array}$$

## 7 Simulation and discussion

**Fig 7:**
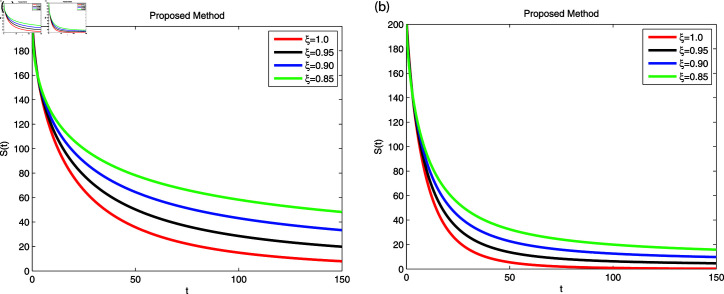
Using Fractal-Fractional operator, the value of *S* ( *t* )  at multiple dimensions with different fractional values (a) 0.6 dimension (b) 0.8 dimension.

**Fig 8:**
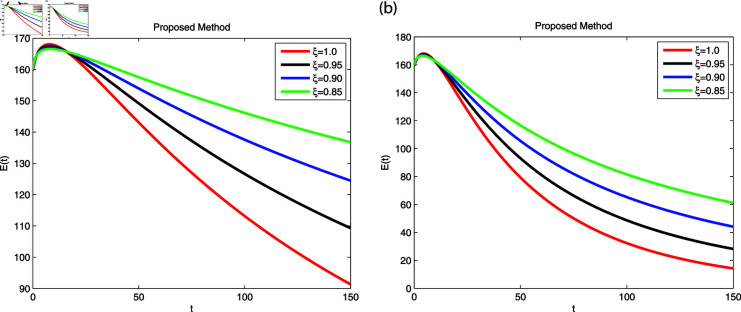
Using Fractal-Fractional operator, the value of *E* ( *t* )  at multiple dimensions with different fractional values (a) 0.6 dimension (b) 0.8 dimension.

**Fig 9:**
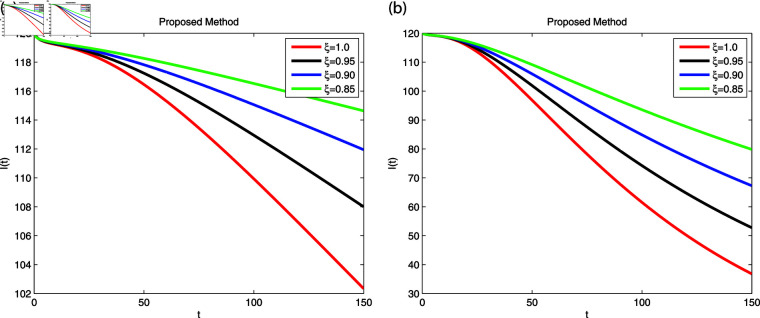
Using Fractal-Fractional operator, the value of *I* ( *t* )  at multiple dimensions with different fractional values (a) 0.6 dimension (b) 0.8 dimension.

**Fig 10:**
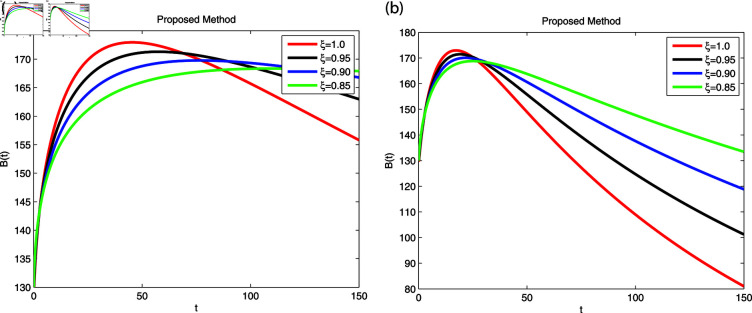
Using Fractal-Fractional operator, the value of *B* ( *t* )  at multiple dimensions with different fractional values (a) 0.6 dimension (b) 0.8 dimension

**Fig 11:**
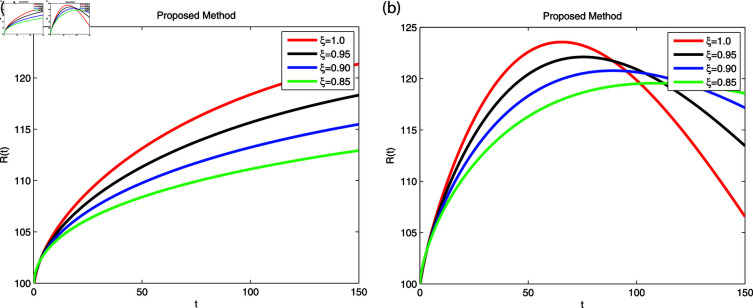
Using Fractal-Fractional operator, the value of *R* ( *t* )  at multiple dimensions with different fractional values (a) 0.6 dimension (b) 0.8 dimension.

We employed the fractal-fractional derivative of the cholera model being connected to the Mittag-Leff1er operator under preset initial conditions to investigate disease transmission employing simulations that comprised both symptomatic and asymptomatic modes of transfer. The simulation data for both dimensions are displayed in Figs 7–11 with different fractional order values 0 . 6and0 . 8. The effectiveness of the obtained theoretical implications is illustrated by a number of cases. Reliable findings are obtained when non-integers parametric possibilities are used for cholera disease, taking consideration of both symptomatic and asymptomatic propagation. Figs 7, 8, 9, 10 and 11 display the outcome for each sub-compartment in dimensions 0 . 66and0 . 8. A computerized simulation for the fractional-order cholera model is generated via MATLAB coding, taking into account both symptomatic and asymptomatic propagation. The parameters values which are adopted from [21], are *μ* = 0 . 01 ,  ∝ = 0 . 00005480 , $\beta_{e}=0.124$ , $\beta_{h}=0.04444$ , *σ* = 0 . 0006 , *α* = 0 . 034 , *ν* = 0 . 02 , *ϕ* = 0 . 029, along with the initial numerical values $S^{0}=200$, $E^{0}=160$, $I^{0}=120$, $B^{0}=130$, and $R^{0}=100$ which are employed in the developed system. Fig 7 depicts the behavior of susceptible people, indicated by *S*. The number of persons grows and then steadies after a brief rapid fall, reaching at steady state at dimensions 0 . 6and0 . 8, respectively. Figs 8, 9, and 10 indicate the exposed peoples, infected people, and the existence of vibrio in the climate. In both scenarios, the population grows at first and steadies at dimensions of 0 . 6and0 . 8, respectively, after a while all sub-compartments approaching at steady state. Fig 7 shows the dynamics of Recovered people (*R*), showing an initial rise, a subsequent decrease, and a point of equilibrium at dimensions of 0 . 6and0 . 8, respectively.

A combination of immune-system-boosting tactics and treatment causes infected persons to fall significantly, as demonstrated in Figs 9, 10 and 11 using various dimensions. It is found that behaviors are similar when utilizing dimensions of either 0.8 or 0.6 with small effects; however, they produce more appropriate outcomes by decreasing dimensions. Additionally, the infected increases due to an increase in Vibrio virus in the environment, as shown in Figs 8a, 9a, and 8b, 9b, respectively. The work makes predictions about the future and offers better strategies for reducing the level of cholera that spreads through the gastrointestinal tract. For all sub-compartments at fractional derivatives, the FFM approach provides superior results than the standard derivatives. It is observed that cholera infected either asymptomatic or symptomatic rises due to vibrio bacterial increase in the environment and approach to stable situation as will as reduces due to control in the stabilization of vibrio bacteria spread in the environment released from cholera infected individuals. Infection can be reduced more effectively by early detection on asymptomatic individuals at acute stage, then automatically symptomatic will reduces, also need to elimination of released infected vibrio bacteria in the environment. Furthermore, it is claimed that lowering dimensions and fractional values improves the dependability and precision of the solutions for every compartment. Researchers may be able to predict what this study can support in the future.

## 8 Conclusion

A fractional-order dynamics cholera disease model that includes asymptomatic cases healed without medical intervention is discussed in this research as well as symptomatic cases including vibrio bacterial infection spread in the environment are under consideration in this investigation. Using a fractal-fractional operator (FFO) with a Mittag-Leff1er kernel for the solutions’ dependability to examined the continuous monitoring of spread of cholera. For those with vigorous immune systems, we offer advice on how to prevent the spread of disease by using asymptomatic measures for early detection, consequently avoiding the need for medication which is helpful for better control measures. The full global impact of the deadly cholera disease with and without symptoms is being examined to see the rate of impact of cholera spread in the environment. In order to verify the created system which is stable or not in a continuous dynamical system, it is necessary to analyzed developed system quantitatively and qualitatively. Next generation technique is utilized to determine the verge condition to observe the rate of spread and also identify that how much parameters are sensitive and we need to maintain the rate of each parameter in specified range. We also examine the results of global efforts to halt the propagation of cholera.

The FFO is utilized to provide accurate and plausible results together with a range of fractional values for ongoing viral spread monitoring. We utilized numerical simulations with MATLAB to witness the real-world dynamics of controlling cholera disease in society by combining asymptomatic and symptomatic interventions to strengthen the immune system and early detection measures. Furthermore, in order to establish control strategies to reduce the threat of cholera in the society, numerical simulation can be used to ascertain the true nature of cholera affects in society using various fractional values. It is being observed that a strong immune system and a combination of treatment modalities are responsible for a rapid elimination of cholera disease, also helpful in reducing the vibrio bacteria release from cholera infected individuals spread in the surrounding environment. On the basis of verified findings, predictions can also be produced for additional study, which will help with early detection and climate diffusion caused by the cholera virus.
